# Elaboration of Charged Poly(Lactic-co-Glycolic Acid) Microparticles for Effective Release of Tranexamic Acid

**DOI:** 10.3390/polym12040808

**Published:** 2020-04-04

**Authors:** Ming-Hsi Huang, Shun-Ying Huang, Yi-Xuan Chen, Cheng-You Chen, Yung-Sheng Lin

**Affiliations:** 1 National Institute of Infectious Diseases and Vaccinology, National Health Research Institutes, Miaoli 35053, Taiwan; huangminghsi@nhri.org.tw; 2 Department of Chemical Engineering, National United University, Miaoli 36063, Taiwan; l0987273913@gmail.com (S.-Y.H.); rosezasx85718@gmail.com (Y.-X.C.); 3 Ph.D. Program in Materials and Chemical Engineering, National United University, Miaoli 36063, Taiwan; wayne20410@gmail.com

**Keywords:** poly(lactic-co-glycolic acid), cetyltrimethylammonium bromide, tranexamic acid, controlled release, charge effect

## Abstract

In this study, tranexamic acid (TA) was used as a model compound to study the charge effect on the physicochemical properties of poly(lactic-co-glycolic acid) (PLGA) microparticles (MPs). Charged PLGA MPs were elaborated by the incorporation of a quaternary ammonium, cetyltrimethylammonium bromide (CTAB), during the double emulsion solvent evaporation process. Three TA-CTAB-carrying modes of PLGA MPs were designed in the CTAB-free (TA-MP), adsorption (TA-CTAB_AD_), or encapsulation (TA-CTAB_EN_) form. The obtained MPs were characterized by morphology and TA-MP affinity. The experiment revealed that the three prepared MPs were spherical and smooth, with pores on their surfaces. TA-CTAB_AD_ had a relatively narrow size distribution, compared with that of TA-MP and TA-CTAB_EN_. The particle sizes of TA-MP, TA-CTAB_EN_, TA-CTAB_AD_ were measured as 59 ± 17, 54 ± 20, and 19 ± 8 μm, respectively. The zeta potential of the three MPs was found to be in the order: TA-CTAB_AD_ > TA-CTAB_EN_ > TA-MP. Differential scanning calorimetry (DSC) indicated that the manufacturing process had no influence on the glass transition temperature of the MPs, which was close to 48 °C. Thermogravimetric analysis illustrated that the presence of CTAB slightly changed the thermal stability of PLGA MPs. *In vitro* release showed that TA-CTAB_AD_ exhibited faster TA release than TA-MP and TA-CTAB_EN_ in a basic environment (pH of 13), probably because of electrostatic attraction. At pH = 1, the release of TA from TA-CTAB_EN_ was faster than those from TA-MP and TA-CTAB_AD_, probably because of electrostatic repulsion. However, the effect of electrostatic interaction was not significant at pH = 7.4.

## 1. Introduction

Poly(lactic-co-glycolic acid) (PLGA) microparticles (MPs) have been widely used as a carrier for the controlled release of candidate drugs [1,2]. The surface charge of PLGA MPs has been utilized for certain applications, such as decreasing plasma protein adsorption [3], carrying genes [4], and targeting cells [5]. It plays a role in various delivery systems in the context of their function on drug delivery, as has been described in the literature [6,7,8,9]. However, the relationship between the elaborating process sequence and drug release ability of charged PLGA MPs remains unclear. Moreover, it is also interesting to investigate the effect of the interior charge of PLGA MPs on their drug release ability. 

Tranexamic acid (TA) with a molecular weight of 157 g/mol, water solubility 1.06 M, and melting point >300 °C [10,11] has been used as an anti-fibrinolytic agent for more than 40 years [12], and was widely used in early medical applications for hemostasis and anti-inflammation. In more recent studies, TA has been shown to interfere with the catalytic reaction of tyrosinase and inhibit melanin production [13,14]. TA is a synthetic derivative of an essential amino acid, lysine. Because TA consists of the –NH_2_ and –COOH groups, the pH of the environment affects the net charge of TA. When the pH of the environment is higher than the isoelectric point (pI), TA tends to be negatively charged, and vice versa. 

Cetyltrimethylammonium bromide (CTAB) is a quaternary ammonium and is commonly used for DNA extraction, as an antiseptic agent against bacteria and fungi, and in household products such as hair conditioner and cosmetics. In the preparation of MPs, CTAB is used as a cationic surfactant due to its positive charge structure. PLGA MPs with CTAB have been used as vaccine vectors to increase the affinity between antigens and MPs through electrostatic attraction. Positively charged MPs effectively adsorb anionic plasmids. This method significantly improves the efficacy of DNA vaccines [15,16]. 

An ideal controlled release should maintain drugs at therapeutically desirable levels. To investigate the charge effect on drug-carrier affinity, we used TA as a model compound to study the release ability from PLGA MPs. The double emulsion method was used to prepare the PLGA MPs with surface charge internal or external to the particles. Finally, the kinetic TA release behaviors from PLGA MPs in different pH environments were analyzed.

## 2. Materials and Methods 

### 2.1. Preparation of PLGA MPs

PLGA 50/50 with an intrinsic viscosity of 0.38 dL/g was purchased from Evonik (Essen, Germany) and used as the carrier substrate. The double emulsion solvent evaporation method was used to prepare PLGA MPs [17]. First, 150 mg of TA was dissolved in 5 mL of 1% (w/v) polyvinyl alcohol (PVA)/water to form the aqueous solution (*W_1_*) and poured into 45 mL of dichloromethane solution containing 450 mg of PLGA and 100 μL of sorbitan trioleate (Span^®^85). The mixture was stirred for 3 min to form the first emulsion (*W_1_/O*), which was then added to a 2000 mL second aqueous solution of 1% (w/v) PVA/water solution (*W_2_*). This mixture was stirred to form the second emulsion (*W_1_/O/W_2_*). For the evaporation of dichloromethane, the second emulsion was stirred continuously for 12 h at room temperature. The hardened MPs were collected through centrifugation, washed twice with deionized water, freeze-dried, and stored in a desiccator before use.

Three TA-CTAB-carrying modes of PLGA MPs were prepared in this study: MPs without CTAB (TA-MP), CTAB-absorbed MPs (TA-CTAB_AD_), and CTAB-encapsulated MPs (TA-CTAB_EN_). TA-MP was used as the control group. For the same 0.5 wt% CTAB content in aqueous solution, TA-CTAB_AD_ was prepared by introducing 10 g of CTAB to *W_2_* in the preparing process, whereas TA-CTAB_EN_ was obtained by adding 25 mg of CTAB to *W_1_* in the process. 

### 2.2. Morphology and Particle Size Distribution

For analyzing morphology and size distribution, the collected freeze-dried PLGA MPs were observed under a scanning electron microscope (Hitachi, TM1000, Tokyo, Japan) and an optical microscope (Olympus, DP70, Tokyo, Japan), respectively. In total, 100 particles were selected from the optical photographs to determine the average diameter of the MPs, and the data are presented as the mean ± standard error (mean ± SEM) of the three samples.

### 2.3. Zeta Potential

The tested sample of PLGA MPs was prepared at a 1:1000 dilution in deionized water. The zeta potential of the samples was measured using Malvern Nano ZS (Malvern Instruments, Malvern, UK) and is presented as mean ± SEM.

### 2.4. Loading Efficiency

According to a previous method [18], 100 mg of freeze-dried PLGA MPs was dissolved in 5 mL of dichloromethane, and 2 mL of deionized water was added to extract TA. The mixture was homogenized using an end-over-end shaker at room temperature. After the aqueous and organic phases were separated, the supernatant was collected, and 2 mL of deionized water was added again for further extraction. The spectrofluorimetric method was adopted to determine the amount of TA in the extraction solution [19]. Subsequently, 100 μL of supernatant extract was mixed with 30 μL 20% (v/v) formaldehyde and 15 µL 8.4% (v/v) acetyl acetone and heated at 95 °C for 10 min. After cooling to room temperature for 20 min, the fluorescence intensity of solution was measured at an excitation wavelength of 415 nm and an emission wavelength of 480 nm. A calibration curve plotted using aliquots of standard TA solutions was used to calculate the concentration of TA in the extraction solution. The TA loading efficiency was calculated using Equation (1):

Loading efficiency (%) = Measured TA/Theoretical TA × 100%(1)

### 2.5. Differential Scanning Calorimetry (DSC) and Thermogravimetric Analysis (TGA)

The Q600 SDT thermal analyzer (TA Instruments, Leatherhead, UK) was used for the thermal analysis: 10 mg of freeze-dried PLGA MPs was measured in platinum pans by heating the samples from room temperature to 105 °C at the rate of 10 °C/min, cooled to 25 °C at 20 °C/min, and reheated to 500 °C at 5 °C/min. All experiments were conducted in a nitrogen atmosphere.

### 2.6. In Vitro Release

To achieve the *in vitro* release, 100 mg of MP was placed in a 5 mL centrifuge tube containing 2 mL of phosphate buffered saline under different pH conditions (pH = 1, 7.4, and 13) in a 37 °C water bath. At predetermined time points, 100 μL samples of the solution were withdrawn from the centrifuge tube to determine the concentration of TA, and 100 μL fresh solutions were filled back to maintain a fixed 2 mL sample volume.

## 3. Results and Discussion

**Figure 1:**
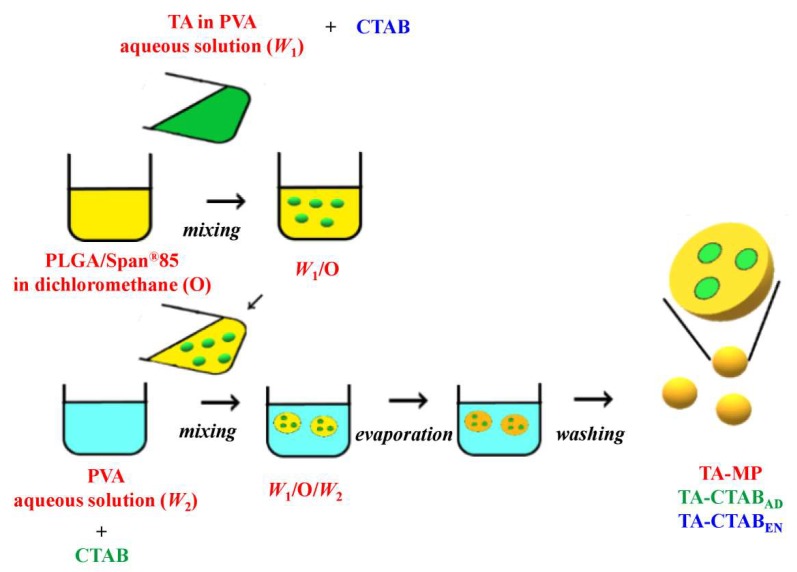
Schematic presentation of the poly(lactic-co-glycolic acid) (PLGA) microparticles (MPs) incorporated with cetyltrimethylammonium bromide (CTAB).

PLGA MPs were prepared using the double emulsion solvent evaporation method described previously [17]. As shown in Figure 1, phase separation occurred at the outset between the aqueous solution and the water-immiscible organic phase; the aqueous solution comprises water and PVA, and the organic phase comprises dichloromethane and PLGA. Following primary emulsification, quasi-stable liquid–liquid dispersion was obtained. However, the emulsion stage lasted only for a few seconds, after which it separated into two layers, indicating thermodynamic instability. Such a heterogeneous emulsion system may encounter problems when bioactive molecules are entrapped within the emulsion. To overcome this problem, Span^®^85 was incorporated in the oily phase to reduce the surface energy at water–dichloromethane interfaces, thus yielding a stable and homogenous emulsion with a dispersed aqueous solution (*W_1_*) and a continuous oily phase (*O*). The resulting primary emulsion (*W_1_/O)* was then dispersed in a second aqueous solution (*W_2_*) containing water and suitable stabilizer PVA to form a double emulsion (*W_1_/O/W_2_*) in the secondary emulsification step. Evaporation of the volatile organic solvent dichloromethane yielded solid MPs. The charge effect can be investigated using the dissolved CTAB either in the internal (*W_1_*) or external aqueous solution (*W_2_*) of the *W_1_/O/W_2_* emulsion; dissolving CTAB in the internal aqueous has the effect of encapsulating CTAB (CTAB_EN_), whereas CTAB-adsorbed MP (CTAB_AD_) can be prepared by dissolving CTAB in *W_2_*. Similarly, TA was loaded by dissolving and mixing in the internal aqueous solution. 

### 3.1. Morphology

**Figure 2:**
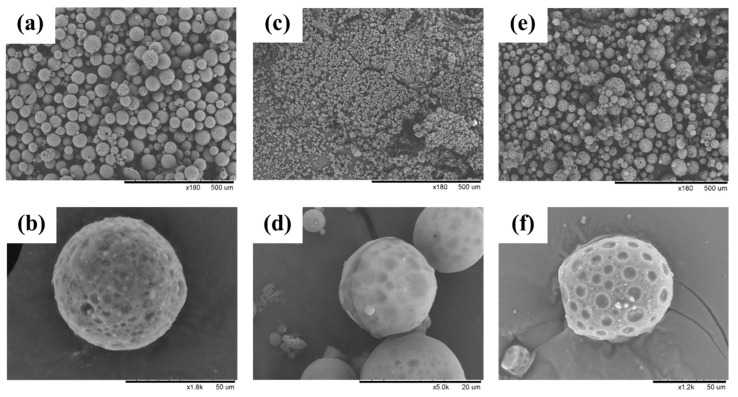
Scanning electron photomicrographs of freeze-dried PLGA MPs: (**a**) tranexamic acid (TA)-MP under low magnification (×180); (**b**) TA-MP under high magnification (×1800); (**c**) TA-CTAB_AD_ under low magnification (×180); (**d**) TA-CTAB_AD_ under high magnification (×5000); (**e**) TA-CTAB_EN_ under low magnification (×180); (**f**) TA-CTAB_EN_ under high magnification (×1200).

The surface morphology of PLGA MPs was observed through scanning electron microscopy. Figure 2 depicts the photomicrographs of the freeze-dried PLGA MPs. The three PLGA MPs were spherical in shape. High-magnification images revealed that the surfaces of the MPs were smooth and that 1–10 μm open pores resulting from rapid solvent extraction in the PLGA hardening process were distributed on the surfaces. The porous surface morphology corresponded to previous studies of PLGA microparticles fabricated using a *W_1_/O/W_2_* double emulsion solvent evaporation technique [20,21,22,23]. This porous surface morphology played an important role in the drug-release process.

### 3.2. Particle Size and Zeta Potential

**Figure 3:**
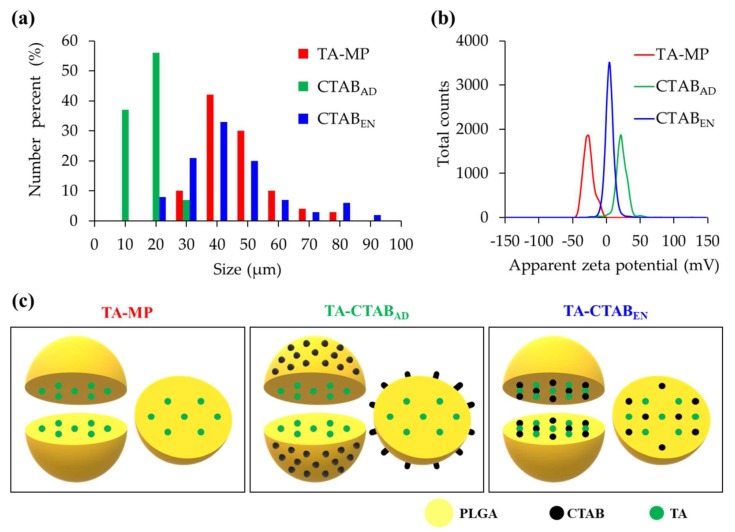
Physicochemical characteristics of the designed PLGA MPs incorporated with TA and CTAB: (**a**) particle size distribution; (**b**) zeta potential; and (**c**) the proposed structure.

The particle size distributions of various PLGA MPs are presented in Figure 3a. The PLGA MPs were not uniform and exhibited a broad-spectrum distribution. The particle sizes of TA-MP and TA-CTAB_EN_ were 59 ± 17 and 54 ± 20 μm, respectively. Compared with the control (TA-MP) and TA-CTAB_EN_, TA-CTAB_AD_ had a relatively narrow particle size distribution. The TA-CTAB_AD_ particles were the smallest among the prepared PLGA MPs, with particle diameters of approximately 19 ± 8 μm. This small size is attributable to the positive charge of the CTAB molecules absorbed on the PLGA MPs. The cationic PLGA emulsified droplets could repel each other, causing less aggregation among the droplets and resulting in their small size. These increasing repulsive interactions can lead to the formation of relatively stable particles with a more uniform size distribution [24].

The zeta potential of PLGA MPs is presented in Figure 3b. The zeta potentials of TA-MP, TA-CTAB_AD_, and TA-CTAB_EN_ were measured −22.9 ± 14.4, 21.4 ± 25.9, and 4.53 ±13.9 mV, respectively. TA-MP had the lowest zeta potential, and the zeta potential of TA-CTAB_AD_ was higher than that of TA-CTAB_EN_. This result is attributable to the CTAB molecules having a positive charge. Consequently, TA-CTAB_AD_ had the highest surface charge and zeta potential. Figure 3c summarizes the proposed structure of PLGA MPs elaborated from different process sequences. 

### 3.3. Loading Efficiency

Per the experimental analysis, the loading efficiency of TA in TA-MP, TA-CTAB_AD_, and TA-CTAB_EN_ were tested as 5.67 ± 1.33%, 5.77 ± 1.49%, and 5.38 ± 0.62%, respectively; the differences were not significant. Interestingly, the presence of CTAB did not affect the loading efficiency of TA in PLGA MPs.

### 3.4. Differential Scanning Calorimetry

**Figure 4:**
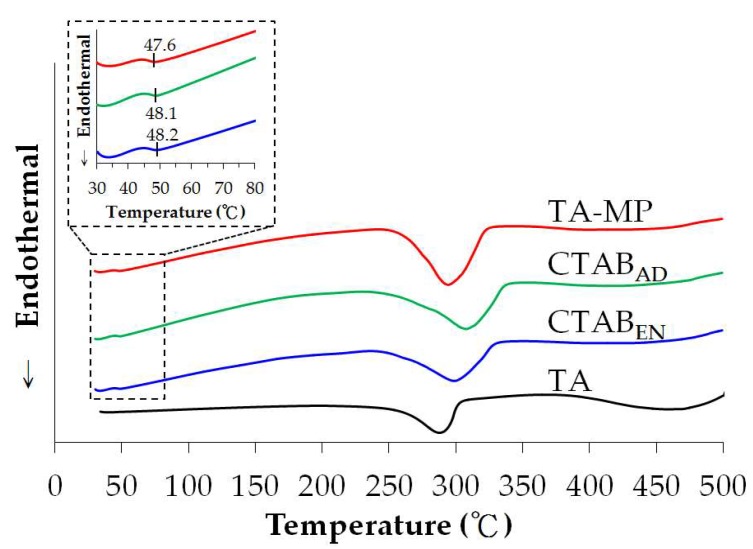
Differential scanning calorimetry (DSC) thermal analysis of TA and various PLGA MPs.

Figure 4 depicts the thermal transitions of PLGA MPs in DSC. Glass transition temperatures (*T_g_*) for TA-MP, TA-CTAB_AD_, and TA-CTAB_EN_ were detected at 47.6, 48.1, and 48.2 °C, respectively. These temperatures are close to those reported *T_g_* of PLGA in the literatures [25,26]. Therefore, *T_g_* were mostly unchanged among the three groups, indicating that the preparation process did not significantly affect the properties of PLGA. Another transition temperature appeared at approximately 300 °C because of the melting of TA and CTAB molecules (approximately 240 °C) [27].

### 3.5. Thermogravimetric Analysis

**Figure 5:**
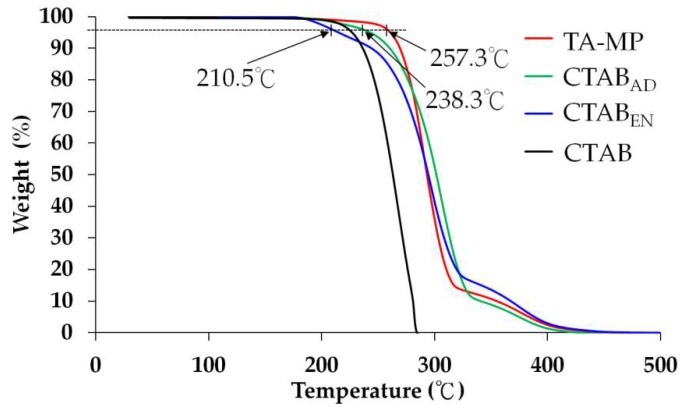
TGA of CTAB and various PLGA MPs.

TGA illustrates the mass change of PLGA MPs as a function of temperature. A material with a low thermal decomposition temperature has low thermal stability. The thermal decomposition temperatures of the PLGA MPs under nitrogen can be obtained from Figure 5. Temperature *T_d_* was defined as the temperature at which the weight loss reached 5%. A *T*_d_ value of 257.3 °C was obtained for the sample TA-MP, and 238.3 °C and 210.5 °C were obtained for the samples TA-CTAB_AD_ and TA-CTAB_EN_, respectively. The TGA curve of the CTAB-containing MPs shifted to a lower temperature compared with that of the CTAB-free PLGA MP, i.e., the overall stability of MPs decreased due to the physical interactions between the CTAB and the PLGA main chain polymer. In fact, it was found that the decomposition of CTAB started at a temperature of 160 °C, a characteristic feature of low-composition temperature that can act as structure-directing agents for the preparation of microporous materials [28]. Additionally, the three PLGA MPs were thermally cracked in two stages. The first crack stage was at approximately 300 °C and the second at approximately 380 °C. It is assumed that the second degradation step was due to the TA content.

### 3.6. In Vitro Release

**Figure 6:**
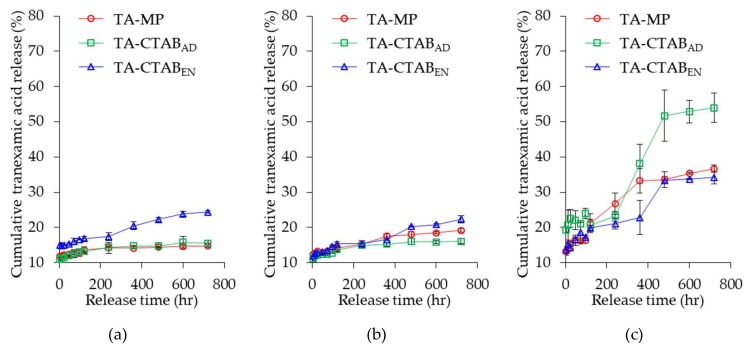
Kinetic release behaviors of TA from various PLGA MPs in different pH environments: (**a**) pH = 1; (**b**) pH = 7.4; (**c**) pH = 13.

The in vitro release of TA was expressed as the measured TA amount in the medium over the initially encapsulated TA amount in the MPs. Figure 6 illustrates the 30-day release behavior of TA from the three PLGA MPs immersed in different solutions (pH = 1, 7.4, and 13). The release rate of PLGA MPs in an alkaline environment was higher than that in acidic and neutral environments. This result is attributable to the faster degradation of PLGA in an alkaline environment [29]. Therefore, the transport barrier was small, and the TA could diffuse out from the PLGA MPs in an alkaline environment [30,31].

The pKa values of carboxylic acid and amine functional groups of TA are 4.9 and 10.6, respectively [32]. Therefore, the pI of TA is 7.3 by the mean of these two pKa values. In pH = 1, TA had a positive charge because its pI was greater than the environmental pH, whereas TA had a negative charge in pH = 13. The repulsive force between positive charges of TA and CTAB in PLGA MPs could accelerate TA release. Therefore, the release rate of TA from TA-CTAB_EN_ was significantly higher than that of TA-MP and TA-CTAB_AD_. In the neutral pH = 7.4 environment with negligible charge effect, the release rates of the three PLGA MPs did not differ significantly. In the alkaline environment (pH = 13), TA had a negative charge, and the attractive force between positive CTAB on the surface of TA-CTAB_AD_ MPs and negative TA in the interior MPs could induce TA to diffuse out. Thus, we infer that the charge effect significantly affected TA release from PLGA MPs.

**Figure 7:**
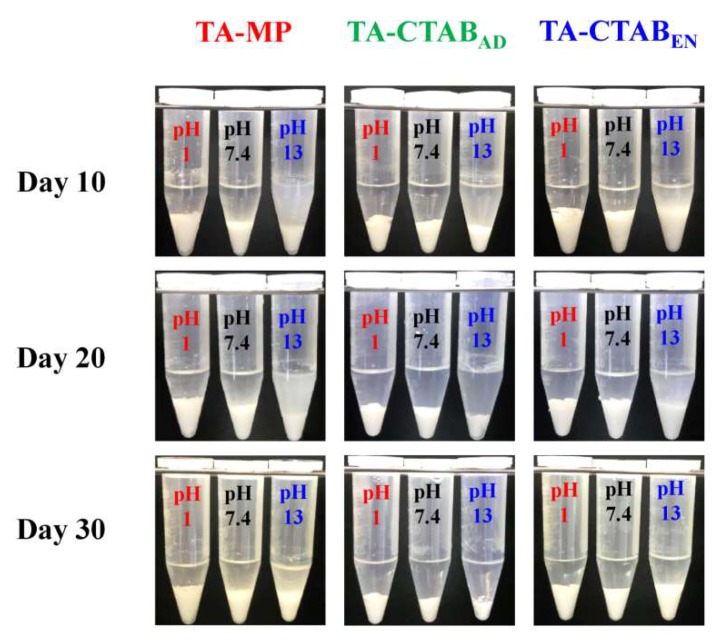
Appearance of the PLGA MPs in three pH solutions (1, 7.4, and 13) for release time points of 10, 20, and 30 days.

Figure 7 depicts the PLGA MPs in three pH solutions at different release time points. The PLGA MPs sank to the bottom because their density was higher than the release solution. The supernatant of the release solution turned turbid at pH 13 on Day 10. However, the supernatant was relatively clear at pH 1 and 7.4. No significant difference was observed in the appearance among the three groups of PLGA MPs. Overall, the dimension of the specimen shrank continuously, indicating the degradation of PLGA follows ester hydrolysis, diffusion, and solubilization of soluble species. 

## 4. Conclusions

This study investigated the charge effect on TA release from PLGA MPs. The three prepared PLGA MPs were spherical and smooth, with pores on the surface. When CTAB was absorbed on the particle surface (TA-CTAB_AD_), the PLGA MPs’ size decreased. Negligible difference was observed in the PLGA microparticle size between the control and CTAB-encapsulated PLGA MPs (TA-CTAB_EN_). The control PLGA MPs had negative zeta potential, whereas PLGA particles with CTAB had positive potential. The zeta potential of CTAB_AD_ was significantly higher than that of CTAB_EN_. DSC and TGA studies indicated that the microparticle manufacturing process had negligible effect on PLGA thermal properties, and PLGA MPs were thermally stable at room temperature. *In vitro* release of TA revealed that PLGA MPs had a high release rate in an alkaline environment. In an acidic environment, the release rate of TA-CTAB_EN_ was significantly higher than that of TA-MP and TA-CTAB_AD_ because of the electrostatic repulsion between TA and CTAB in the MPs. The electrostatic attraction of surface CTAB on TA in MPs induced TA release for TA-CTAB_AD_ in the alkaline environment. However, no significant difference was observed in the release rate of the PLGA MPs in the neutral environments.

## References

[1] Danhier F., Ansorena E., Silva J.M., Coco R., Le Breton A., Préat V. (2012). PLGA-based nanoparticles: An overview of biomedical applications. J. Control. Release.

[2] Martin-Banderas L., Durán-Lobato M., Muñoz-Rubio I., Alvarez-Fuentes J., Fernández-Arevalo M., Holgado M.A. (2013). Functional PLGA NPs for oral drug delivery: Recent strategies and developments. Mini Rev. Med. Chem..

[3] Shubhra Q.T.H., Tóth J., Gyenis J., Feczkó T. (2014). Surface modification of HSA containing magnetic PLGA nanoparticles by poloxamer to decrease plasma protein adsorption. Colloids Surf. B Biointerfaces.

[4] Ravi-Kumar M.N., Bakowsky U., Lehr C.M. (2004). Preparation and characterization of cationic PLGA nanospheres as DNA carriers. Biomaterials.

[5] Radovic-Moreno A.F., Lu T.K., Puscasu V.A., Yoon C.J., Langer R., Farokhzad O.C. (2012). Surface charge-switching polymeric nanoparticles for bacterial cell wall-targeted delivery of antibiotics. ACS Nano.

[6] Acharya S., Sahoo S.K. (2011). PLGA nanoparticles containing various anticancer agents and tumour delivery by EPR effect. Adv. Drug Deliv. Rev..

[7] Hamdy S., Haddadi A., Hung R.W., Lavasanifar A. (2011). Targeting dendritic cells with nano-particulate PLGA cancer vaccine formulations. Adv. Drug Deliv. Rev..

[8] Honary S., Zahir F. (2013). Effect of zeta potential on the properties of nano-drug delivery systems—A review (Part 1). Trop. J. Pharm. Res..

[9] Honary S., Zahir F. (2013). Effect of zeta potential on the properties of nano-drug delivery systems—A review (Part 2). Trop. J. Pharm. Res..

[10] Haghi M., van den Oetelaar W., Moir L.M., Zhu B., Phillips G., Crapper J., Young P.M., Traini D. (2015). Inhalable tranexamic acid for haemoptysis treatment. Eur. J. Pharm. Biopharm..

[11] Vijayakumar A., Baskaran R., Yoo B.K. (2017). Skin permeation and retention of topical bead formulation containing tranexamic acid. J. Cosmet. Laser Ther..

[12] Gibbs J.R., Corkill A.G. (1971). Use of an anti-fibrinolytic agent (tranexamic acid) in the management of ruptured intracranial aneurysms. Postgrad. Med. J..

[13] Kanechorn Na Ayuthaya P., Niumphradit N., Manosroi A., Nakakes A. (2012). Topical 5% tranexamic acid for the treatment of melasma in Asians: A double-blind randomized controlled clinical trial. J. Cosmet. Laser Ther..

[14] Tse T.W., Hui E. (2013). Tranexamic acid: An important adjuvant in the treatment of melasma. J. Cosmet. Dermatol..

[15] O’Hagan D.T., Valiante N.M. (2003). Recent advances in the discovery and delivery of vaccine adjuvants. Nat. Rev. Drug Discov..

[16] O’Hagan D.T., Singh M., Ulmer J.B. (2004). Microparticles for the delivery of DNA vaccines. Immunol. Rev..

[17] Mundargi R.C., Babu V.R., Rangaswamy V., Patel P., Aminabhavi T.M. (2008). Nano/micro technologies for delivering macromolecular therapeutics using poly(D,L-lactide-co-glycolide) and its derivatives. J. Control. Release.

[18] Cun D., Jensen D.K., Maltesen M.J., Bunker M., Whiteside P., Scurr D., Foged C., Nielsen H.M. (2011). High loading efficiency and sustained release of siRNA encapsulated in PLGA nanoparticles: Quality by design optimization and characterization. Eur. J. Pharm. Biopharm..

[19] El-Aroud K.A., Abushoffa A.M., Abdellatef H.E. (2007). Spectrophotometric and spectrofluorimetric methods for the determination of tranexamic acid in pharmaceutical formulation. Chem. Pharm. Bull..

[20] Amoyav B., Benny O. (2019). Microfluidic based fabrication and characterization of highly porous polymeric microspheres. Polymers.

[21] Icart L.P., Souza F.G., Lima L.M.T.R. (2019). Sustained release and pharmacologic evaluation of human glucagon-like peptide-1 and liraglutide from polymeric microparticles. J. Microencapsul..

[22] Kuriakose A.E., Hu W., Nguyen K.T., Menon J.U. (2019). Scaffold-based lung tumor culture on porous PLGA microparticle substrates. PLoS ONE.

[23] Biswal A.K., Hariprasad P., Saha S. (2020). Efficient and prolonged antibacterial activity from porous PLGA microparticles and their application in food preservation. Mater. Sci. Eng. C.

[24] Hans M.L., Lowman A.M. (2002). Biodegradable nanoparticles for drug delivery and targeting. Curr. Opin. Solid State Mater. Sci..

[25] Passerini N., Craig D.Q. (2001). An investigation into the effects of residual water on the glass transition temperature of polylactide microspheres using modulated temperature DSC. J. Control. Release.

[26] Mu L., Feng S.S. (2003). A novel controlled release formulation for the anticancer drug paclitaxel (Taxol): PLGA nanoparticles containing vitamin ETPGS. J. Control. Release.

[27] Attwood D., Elworthy P.H., Kayne S.B. (1970). Membrane osmometry of aqueous micellar solutions of pure nonionic and ionic surfactants. J. Phys. Chem..

[28] Huang L., Chen X., Li Q. (2001). Synthesis of microporous molecular sieves by surfactant decomposition. J. Mater. Chem..

[29] Li J., Jiang G., Ding F. (2008). The effect of pH on the polymer degradation and drug release from PLGA-mPEG microparticles. J. Appl. Polym. Sci.

[30] Singh V., Singh S., Das S., Kumar A., Self W.T., Seal S. (2012). A facile synthesis of PLGA encapsulated cerium oxide nanoparticles: Release kinetics and biological activity. Nanoscale.

[31] Sousa F., Cruz A., Fonte P., Pinto I.M., Neves-Petersen M.T., Sarmento B. (2017). A new paradigm for antiangiogenic therapy through controlled release of bevacizumab from PLGA nanoparticles. Sci. Rep..

[32] Grassin Delyle S., Abe E., Batisse A., Tremey B., Fischler M., Devillier P., Alvarez J.C. (2010). A validated assay for the quantitative analysis of tranexamic acid in human serum by liquid chromatography coupled with electrospray ionization mass spectrometry. Clin. Chim. Acta.

